# Harnessing Natural Recovery Processes to Improve Restoration Outcomes: An Experimental Assessment of Sponge-Mediated Coral Reef Restoration

**DOI:** 10.1371/journal.pone.0064945

**Published:** 2013-06-04

**Authors:** Brendan C. Biggs

**Affiliations:** Department of Biological Science, Florida State University, Tallahassee, Florida, United States of America; Leibniz Center for Tropical Marine Ecology, Germany

## Abstract

**Background:**

Restoration is increasingly implemented to reestablish habitat structure and function following physical anthropogenic disturbance, but scientific knowledge of effectiveness of methods lags behind demand for guidelines. On coral reefs, recovery is largely dependent on coral reestablishment, and substratum stability is critical to the survival of coral fragments and recruits. Concrete is often used to immobilize rubble, but its ecological performance has not been rigorously evaluated, and restoration has generally fallen short of returning degraded habitat to pre-disturbance conditions. Fragments of erect branching sponges mediate reef recovery by facilitating rubble consolidation, yet such natural processes have been largely overlooked in restoring reefs.

**Methods:**

On two reefs in Curacao, four treatments - coral rubble alone, rubble seeded with sponge fragments, rubble bound by concrete, and concrete “rubble” bound by concrete - were monitored over four years to investigate rubble consolidation with and without sponges and the ecological performance of treatments in terms of the number and diversity of coral recruits. Species specific rates of sponge fragment attachment to rubble, donor sponge growth and tissue replacement, and fragment survival inside rubble piles were also investigated to evaluate sponge species performance and determine rates for sustainably harvesting tissue.

**Findings/Significance:**

Rubble piles seeded with sponges retained height and shape to a significantly greater degree, lost fewer replicates to water motion, and were significantly more likely to be consolidated over time than rubble alone. Significantly more corals recruited to sponge-seeded rubble than to all other treatments. Coral diversity was also greatest for rubble with sponges and it was the only treatment to which framework building corals recruited. Differences in overall sponge species performance suggest species selection is important to consider. Employing organisms that jump start successional pathways and facilitate recovery can significantly improve restoration outcomes; however, best practices require techniques be tailored to each system.

## Introduction

Coral reefs harbor astonishing biodiversity and provide numerous ecosystem goods and services vital to the economies of tropical and sub-tropical coastal nations [1]–[3]. Rapid and persistent declines in coral reef health, particularly in the Caribbean [4]–[7], have drawn attention to the consequences of anthropogenic activities and spurred interest in reef conservation and management (e.g., [6], [8]–[10]). Where reefs have been degraded or lost due to physical anthropogenic disturbances (e.g., vessel groundings, anchor dragging and dynamite fishing), rehabilitation and restoration have often been employed to reestablish habitat structure and function [11]. Given that natural recovery can take decades or even centuries [12]–[16], and that worldwide reef health is in decline due to the increasing frequency of anthropogenic disturbances (e.g., [3], [7]), active intervention following injury is not only warranted but may increasingly be required [11]. However, the practice of reef restoration is in its infancy [17], and scientific knowledge of the effectiveness of various methods has often lagged behind demand for guidelines [11].

Most restoration efforts have focused on repopulating reefs with transplants of coral fragments or whole colonies and on reestablishing substratum stability [17]. Generating stable substrata can include binding together fractured framework and unstable debris with concrete, as well as removing coral rubble and replacing it with concrete blocks, domes, or mats [11], [18]. Given that reef recovery is largely dependent on coral reestablishment, and that substratum stability is critical to the survival of coral fragments and recruits [19]–[23], such activities seem appropriate; nevertheless, these considerable efforts have fallen short of returning degraded reef habitat to pre-disturbance conditions [11], [24]. The fundamental difficulty of trying to reconstruct complex systems [25] coupled with deteriorating conditions in marine environments [10], [26] have likely contributed to the lack of success. However, failure to restore damaged reefs may also reflect our general lack of knowledge of what works or does not work and why [11].

Though it is commonly accepted that restoration works best when the physical environment is “gotten right” [27], [28], rigorous evaluation of the ecological performance of artificial materials and their alternatives have largely not been made ([24], but see [29], [30]). As a result, the attractiveness of such materials to coral larvae in general, and framework building corals in particular, remains questionable. Patterns of preferential recruitment to natural substrata (e.g., [29], [31]–[34]) suggest that while corals recruit to artificial surfaces [29], [35], their abundance and diversity at restoration sites could be increased by greater use of natural materials (i.e., substrata and binding agents). Restoration success might be further improved by employing successional processes [28] that naturally generate stable substrata [20], [36,]; however, natural processes facilitating rubble consolidation have been largely overlooked in reef restoration practice.

Facilitators have been successfully used in a variety of habitats to improve restoration outcomes (e.g., [37]–[40]), and sponges may be able to do the same for coral reefs [36], [41]. Natural consolidation of coral rubble (binding together and to the reef) occurs through the growth of carbonate secreting organisms, such as crustose coralline algae (CCA), and by diagenetic cementation (reviewed by [42]). However, rubble too light to remain stationary must first be stabilized, or the process of consolidation may be continually disturbed by water motion and bioturbation [20], [42]. Fragments of erect branching coral reef sponges mediate rubble consolidation by temporarily stabilizing rubble [20]. Firm attachment to rubble and the reef by sponges allows time for the settlement and growth of carbonate secreting organisms, which rigidly bind rubble together and to the reef. Experimental assessment of these interactions on shallow reefs in Panama revealed that the entire sequence of events, from the temporary stabilization of rubble by sponges to the consolidation of rubble by CCA and the arrival of coral recruits, can occur in as little as ten months [20]. Additionally, chemical cues emitted by species of CCA have been shown to attract coral larvae and induce metamorphosis [reviewed by 43], and corals that recruited to CCA in the Indo-Pacific had greater survival and faster growth [44]. Sponge allelochemicals have also been found to attract the larvae of benthic invertebrates [45], and branching sponges preempt little area from coral recruitment due to their erect growth form and small base to volume ratios. Asexual propagation dominates the life histories of branching sponges [46], [47], and the ability of fragments to survive separation and reattach rapidly to the benthos with any part of the existing sponge material [48] make them ideal candidates for use in stabilizing coral rubble. Furthermore, high survival of sponge fragments [46], [47] suggests their use would be non-consumptive, and essentially constitute propagation. In total, this evidence suggests that, while restoration efforts employing artificial agents have been successful in producing stable substrata, incorporating natural materials and ecological interactions into methods currently used to restore coral reefs could be highly beneficial.

Using three species of erect branching sponges, this study addressed the following four questions: 1) can sponge fragments be used to generate stable, natural substrata; 2) how well does rubble seeded with sponge fragments perform as recruitment substrata relative to coral rubble without sponges, concrete bound coral rubble, and concrete bound concrete “rubble”; 3) at what rate can tissue be sustainably harvested from sponges for use in seeding rubble; and 4) are there species specific differences in sponge attachment, growth, and tissue replacement rates as well as fragment performance in rubble piles such that species selection must be considered?

## Materials and Methods

### Study Sites and Sponge Species

**Figure 1:**
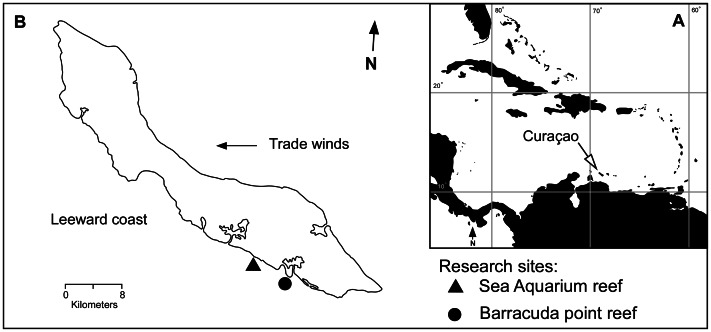
Location of study sites. A. Map of the Caribbean with the location of Curaçao indicated. B. Map of Curaçao, Netherlands Antilles. Filled triangle and circle indicate location of study sites Sea Aquarium reef and Barracuda Point reef, respectively.

The rubble consolidation and coral recruitment experiments reported here were conducted on two fringing reefs along the south-east coast of Curaçao, in front of the Curaçao Sea Aquarium (**SA**) (12° 5′ 0.68″ N, 68° 53′ 40.81′ W) and eastward along the coast at Barracuda Point (**BP**) (12° 3′ 44.91″N, 68° 51′ 22.35″ W) (Figure 1). Growth and tissue replacement rates of donor sponges were investigated at BP, as were rates of attachment of individual sponge fragments to single pieces of coral rubble (Figure 1B). Using these sites to address our questions is meaningful from a management perspective in that fringing reefs form in relatively shallow coastal waters where boat traffic and recreational activities increase their risk of physical disturbance [49]–[51]. Furthermore, these sites allow examination of sponge stabilizing performance and rubble pile consolidation under different degrees of water motion. Atlantic and Gulf Rapid Reef Assessment surveys (AGRRA 2005, v. 4.0) conducted prior to the study revealed similarity in coral composition (Tables S1 and S2) between the sites but difference in substratum composition (Figure S1): mobile substrata (e.g., coral rubble and sand) accounted for 35% of substrata at BP but only 15% at SA. Reduced accumulation of mobile substrata on the benthos at SA suggests greater intensity of water motion at this site, which can disrupt consolidation of loose rubble. Additional sources of disturbance include tropical cyclones, which pass within 180 nmi of Curaçao at a frequency of roughly 0.39 storms per year [52]. While wave heights from 0.3 to 1.5 m are typical year round along the south-east coast, average heights may be exceeded during the hurricane season [53].

**Figure 2:**
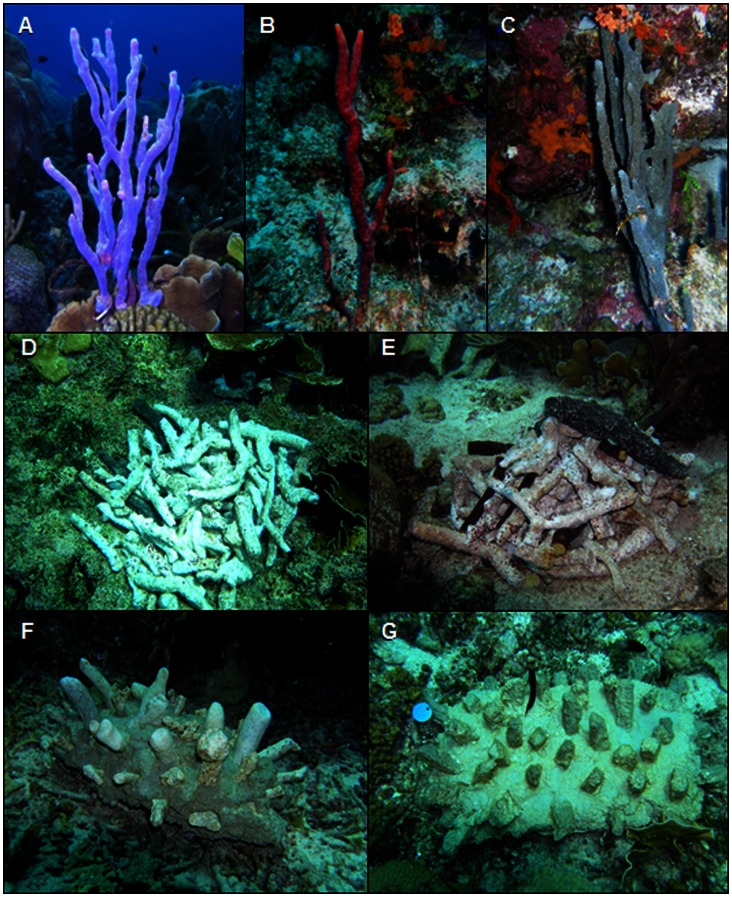
Erect branching sponge species and experimental substratum treatments. A. *Aplysina cauliformis.* B. *Aplysina* sp. C. *Niphates erecta.* D. Coral rubble alone. E. Coral rubble with sponge fragments inserted into pile. F. Concrete bound coral rubble. G. Concrete bound concrete “rubble”.

Three sponge species, *Aplysina cauliformis* (Carter, 1882), *Aplysina* species, and *Niphates erecta* (Duchassaing and Michelotti, 1864) (Figure 2A–2C) were selected for testing based on their shared erect branching morphology and relative abundance at each site. *Aplysina cauliformis* and *N. erecta* are common and abundant members of shallow water reef communities throughout the Caribbean. *Aplysina* species also appears to be broadly distributed, though, while abundant in Curaçao, it is usually found in low numbers at most sites (personal observation).

### Treatments Used to Investigate Rubble Stabilization and Consolidation and Coral Recruitment

Four treatments varying in substratum and binding agent were constructed and deployed at each reef site in 2007 (July and October for SA and BP, respectively). Treatments (Figure 2D–2G) included: 1) coral rubble alone; 2) coral rubble seeded with sponge fragments; 3) coral rubble bound by concrete, and 4) “rubble” made of concrete bound by concrete (N = 21 and 20 replicates of each treatment at SA and BP, respectively). At each site, treatments were grouped to form “clusters”, each cluster containing one representative from each of the four treatments. Under the assumption that water motion decreases with increasing depth, clusters were positioned such that they were stratified among three depths (shallow, intermediate and deep) (Table 1).

**Table 1:** Number of treatment clusters by site and depth.

		Depth stratum		
	Total	Shallow(2–4 m)	Intermediate(4–6 m)	Deep(4–8 m)
**Study Sites**	**(N)**	**(N)**	**(N)**	**(N)**
Sea Aquarium	21	6	11	4
Barracuda Point	20	2	13	5

Each treatment cluster contained one replicate from each of the four treatments (Figure 2D–2G).

All rubble piles measured 30 cm in diameter and 20 cm in height initially, and were formed from sun-bleached skeletons of *Acropora cervicornis*. On average, 43.9+/−5.9 (mean +/− SD) pieces of coral rubble measuring 21.25+/−8.6 cm in length and 2.73+/−1.1 cm in width were used to form each replicate pile. Volume of rubble per pile was standardized by filling a single plastic bin (35 cm in length and width, 18 cm in height) with rubble to form each pile; bins were also used to transport rubble to assigned locations.

To form each sponge-rubble replicate pile, 8 pieces of coral rubble were removed from an established pile and a single 10 cm long sponge fragment was secured to each (N = 8 sponge fragments per pile) using thin nylon cable ties. These rubble-sponge units were then haphazardly inserted into and laid on top of the pile such that initial pile dimensions were achieved. Seeding all sponge-rubble piles required 328 branch tips, excised with razor blades from N = 128, 168, and 32 healthy individuals of *A. cauliformis*, *Aplysina* sp., and *N. erecta,* respectively. Before use, fragments were allowed to heal for 48 hours in plastic “berry baskets” stored in mesh dive bags anchored to the benthos. Latex gloves were worn while working with sponges and fragments were never exposed to air. Relative sponge abundance at each site determined the number of rubble piles seeded with any one species (Table 2).

**Table 2:** Number of sponge-rubble piles by site seeded with fragments of each sponge species.

	Sea Aquarium		Barracuda Point	
Sponge species	(N)	(%)	(N)	(%)
*Aplysina cauliformis*	11	81.8	5	80
*Aplysina* sp.	8	37.5	13	53.8
*Niphates erecta*	0	–	2	100
Mixture	2	50	0	–

Percent of piles whose fragments survived to month 48 at Sea Aquarium and 45 at Barracuda Point are also given.

Mixture refers to piles that received a combination of *A. cauliformis* and *Aplysina* sp. fragments.

Replicate concrete-rubble and concrete-concrete treatments were constructed on land on top of clean limestone sand. For the concrete-rubble treatment, pieces of sun-bleached coral rubble (one plastic bin full per replicate structure) were inserted into a wet base layer (∼6 cm thick and ∼30 cm in length and width) of concrete (one part Portland type 2 cement to three parts pulverized coral rock) such that pieces protruded vertically, obliquely, and horizontally. Structure design was intended to increase three-dimensional complexity in order to provide a variety of micro habitats [54]. Concrete-concrete replicates were constructed similarly, except that “rubble” inserted into the concrete was formed from concrete. Concrete “rubble” pieces were of similar dimensions as coral rubble. All concrete bound structures were allowed to cure for 5 days before deployment.

Dimensions of all replicate concrete-rubble and concrete-concrete structures mirrored those of rubble-alone and sponge-rubble piles; thus, per-replicate surface area available for recruitment was roughly equal among replicates and therefore treatments: 5,149.4+/−621.52 cm^2^ (mean +/− SD) per rubble pile (for both rubble-alone and sponge-rubble treatments); 4,829.8+/−582.9 cm^2^ per concrete-rubble structure; and 5,417.2+/−344.1 cm^2^ per concrete-concrete “rubble” structure. Surface area available for recruitment was calculated for rubble-alone and sponge-rubble assuming one-half of all rubble surfaces were exposed after pile formation. Once positioned on the reef, numbered aluminum tags were secured to the benthos next to each pile/structure for identification purposes.

### Stability and Consolidation of Rubble Piles with and without Sponges

Stability of rubble-alone and sponge-rubble piles was quantified in terms of height [cf. 20]; thus a direct positive relationship between stability and height was assumed. Pile height relative to the substratum was measured during roughly annual surveys: months 12, 24, 36, and 48 after deployment at SA, and months 12, 21, 33, and 45 at BP. Except for month 12 at BP, surveys at each site were conducted each year in July. During each survey piles were also visually inspected for the presence of stabilizing (e.g., turf and macro algae, cryptic sponges) and consolidating organisms (e.g., CCA, bryozoans and hydrocorals); the condition of sponge fragments inserted into sponge-rubble piles was also assessed at this time. Lastly, all piles were subjected to hand pressure (probing and prodding) as in Hudson and Diaz [55], to determine consolidation. Piles in which 90% or more of rubble pieces did not move due to encrustation by carbonate secreting organisms were considered consolidated.

Differences in heights of rubble piles at each site were analyzed using linear mixed-effects models (LME) (R package nlme [56]; Gaussian distribution) with water depth (shallow, intermediate and deep) and treatment (rubble-alone and sponge-rubble) as fixed effects in the maximal model. Replicates nested within time were used as a random effect to account for repeated measures of height for individual rubble piles. Pairwise comparisons of means with Bonferroni correction were used to determine differences between levels of fixed effects. G-tests of independence were used to analyze differences in the proportion of consolidated vs. unconsolidated piles among treatments (rubble-alone, sponges-rubble) at each time period, for each site, respectively.

### Coral Recruitment to Rubble Piles with and without Sponges and Concrete Bound Structures

During each annual survey, corals that had recruited to experimental substrata were enumerated, identified to lowest taxonomic rank, mapped and photographed. Coral recruits were surveyed *in situ*, thus corals recruiting to the insides of rubble piles were likely missed; recruitment to rubble-alone and sponge-rubble treatments are therefore conservative estimates. Differences among treatments in the number of coral recruits were investigated separately for each site in two ways: 1) generalized liner models (GLM) (R package MASS [57], Poisson distribution) were used to analyze the number of coral recruits present at months 48 and 45 at SA and BP, respectively; and 2) LME (R package nlme; Gaussian distribution) were used to investigate differences in recruitment among treatments over time at each site. Treatment (rubble alone, rubble seeded with sponges, rubble bound by concrete, concrete “rubble” bound by concrete) and depth (shallow, intermediate, deep) were included as factors or fixed-effects in the GLM’s and LME’s, respectively. Depth did not significantly influence numbers of coral recruits at either site over time, or at months 48 and 45, and was therefore not retained in any minimal adequate model. Pairwise comparisons of means with Bonferroni correction were used to determine differences between significant factor levels or fixed effects for GLM’s and LME’s, respectively.

### Comparison of Fragment Attachment Rate among Sponge Species

Insertion of sponge fragments into rubble piles hindered direct observation of attachment; thus, a separate experiment was used to quantify the rate at which fragments of each species attach to coral rubble. Single branch tips (8 cm in length) were excised with razor blades in July 2007 from 11, 19, and 12 individuals of *A. cauliformis*, *Aplysina* sp., and *N. erecta*, respectively at BP. Individuals were selected haphazardly from those between 4 and 9 m of depth. Each fragment was secured to a single piece of clean coral rubble using a thin nylon cable tie such that fragments were able to move but would not be swept away by water motion. Fragments were measured (volume), tagged with numbered Floytags, and arranged haphazardly on the benthos. Attachment was checked daily, and determined by visually inspecting the interface between sponge and rubble for tissue growth and by gently probing fragments to assess movement. Preliminary analysis via analysis of covariance (ANCOVA) revealed no influence (interaction or main effect) of fragment volume on attachment rate. Number of days taken by fragments to attach was therefore analyzed using analysis of variance (ANOVA), with sponge species as a factor (*A. cauliformis*, *Aplysina* sp., *N. erecta*).

### Growth of and Tissue Replacement by Donor Sponges

Rates at which sponge tissue can be sustainably harvested from individuals to seed coral rubble were investigated in two ways: 1) by measuring growth; and 2) by measuring the replacement of excised tissue over time. Following an initial faunal survey at BP (July, 2007), a total of 59, 60, and 61 individuals of *A. cauliformis*, *Aplysina* sp. and *N. erecta*, respectively were haphazardly selected. Each individual was tagged, mapped and photographed to facilitate relocation and identification. To determine initial size and investigate growth, the total volume of each individual was measured in detail (by geometric approximation as in [41]) in July, 2007 and 2008. To investigate the replacement of tissue excised (in terms of volume) to seed rubble piles, a 10 cm length of tissue was harvested from the most distal portion of a single erect branch of each individual; the same individuals were used to study growth and tissue replacement. Each excised fragment was measured and the volume of tissue removed recorded. Since branches add tissue at their tips, a thin nylon cable tie was affixed to each cut branch 2 cm below the point of excision to aid accurate measurement of tissue replacement. Volume of tissue replaced by each branch tip was measured at 3 month intervals for 15 months.

Growth was calculated as annual percent change in volume (**APC** = [V_Final_ – V_Initial_/V_Initial_]×100), where V_Initial_ and V_Final_ are the volumes of individuals as measured in July, 2007 and 2008, respectively. Given that a rate of tissue production was sought, and that fragmentation is common among erect branching sponges [47], APC was only calculated for individuals that increased in size over the 12 month period. Growth calculations are therefore conservative, as it is likely even those sponges that increased in size lost tissue through fragmentation. Tissue replacement was calculated as the percent of excised tissue that had been replaced, in terms of volume, at each survey period **(PVR** = [V_t_/V_x_]×100). For PVR, V_t_ is the volume of tissue replaced measured at t = 3, 6, 9, 12, and 15 months after excision, and V_x_ the volume of tissue excised.

Annual percent change (APC) was analyzed using ANOVA, with species as a factor (*A. cauliformis*, *Aplysina* sp., and *N. erecta*). Log transformed initial sponge size was not included as a covariate in the minimal adequate model given that preliminary analysis revealed no significant interaction and no significant effect of log transformed initial size on APC. Differences in PVR were analyzed using LME (R package nlme [56]; Gaussian distribution), with sponge species (*A. cauliformis*, *Aplysina* sp., *N. erecta*), log transformed initial sponge volume, and the interaction between the two fixed effects in the maximal model. Individual sponges were included as a random effect to account for repeated measures of PVR. Pairwise comparisons of means with Bonferroni correction were used to determine differences between levels of significant main effects for APC and fixed effects for PVR. Water depth was not included in any of the analyses as preliminary investigation revealed no significant correlation between APC or PVR and depth for any species.

### Statistical Analyses

All analyses were conducted in R, version 2.14.1 (R Development Core Team 2007) running on a Windows platform. Statistical assumptions of all models used were tested through graphical analysis of the residuals. Following Zurr et al. [58], a top down model selection process was conducted whereby nested models were compared using likelihood ratio tests, Chi squared tests of deviance, and Akaike information criterion, depending on the model, to determine each minimally adequate model. Repeated measures data sets were analyzed with LME’s, as they allow for testing of fixed effects using appropriate degrees of freedom [58], [59]. For each LME, the optimal structure of the random components was found under restricted maximum likelihood estimation (REML), after which the optimal fixed structure was found under maximum likelihood estimation (ML); minimal adequate models are presented using REML. During LME random components optimization, a correlation structure was included in the model if autocorrelation plots of normalized residuals suggested violation of independence; a variance structure that allowed for different variance per stratum was included if plots of normalized residuals vs. explanatory variables revealed heterogeneity of variance [58] (See Text S1 for further details).

### Ethics Statement

No species, protected or otherwise, were sampled, collected, or transported during the course of this study. The study was conducted at a time when approval from an official body was not required for manipulations of sponges; therefore, no permits were required. Manipulation of erect branching sponges is nondestructive and minimally invasive given that these sponges fragment naturally and heal cut surfaces rapidly. Research was conducted through the Curacao Sea Aquarium, who granted shore access to the reef in front of their facility, and CARMABI, who authorized continued observation of experiments. Shore access to Barracuda Point was available via public lands.

## Results

### Stability and Consolidation of Rubble Piles with and without Sponges

**Figure 3:**
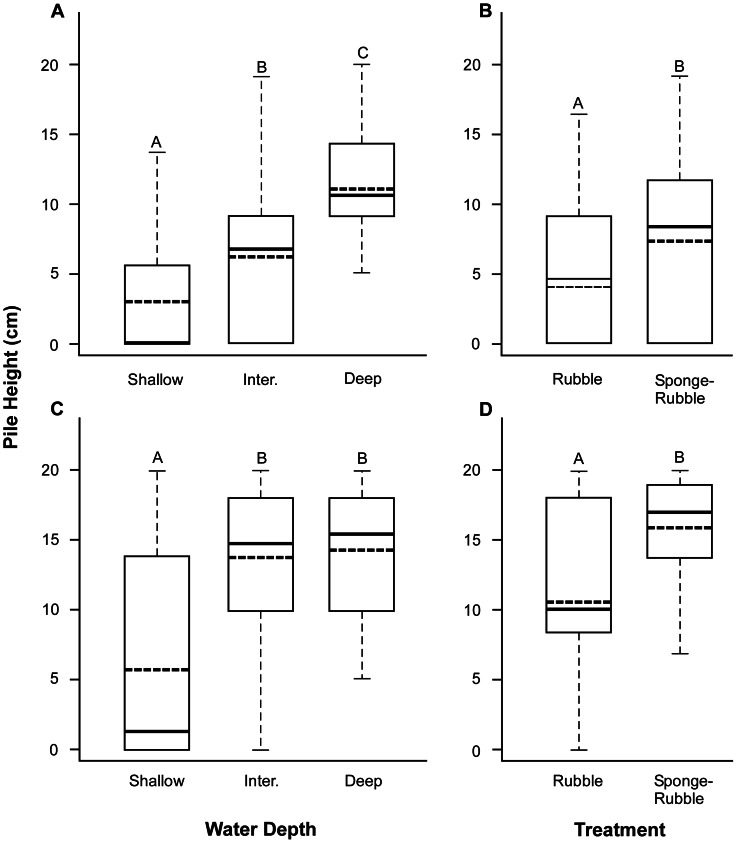
Influence of treatment and water depth on rubble pile height over time. Conditional boxplots of rubble pile height for Sea Aquarium and Barracuda point. A and C. Rubble pile height conditional on water depth at Sea Aquarium and Barracuda Point, respectively. B and D. Rubble pile height conditional on treatment at Sea Aquarium and Barracuda Point, respectively. Thick solid and broken bars inside boxes indicate median and mean height, respectively. Letters above boxes indicate significant differences (P<0.05) between factor levels based on pairwise comparison of means with Bonferroni correction.

At both sites treatment significantly influenced rubble pile height over time (LME, F _1, 38_ = 4.77, P = 0.035, and LME, F _1, 36_ = 14.37, P = 0.0006 for SA and BP, respectively), and temporary stabilization of coral rubble by sponges resulted in significantly greater retention of initial pile height (Figure 3B and 3D). Water depth also influenced rubble pile height at each site over time (LME, F _2, 39_ = 13.39, P<0.0001, and LME, F _2, 36_ = 6.88, P = 0.003 for SA and BP, respectively). As expected if water motion decreases with increasing water depth, pile height at SA was positively related to depth, and mean height differed significantly between each depth stratum (Figure 3A). A similar trend was noted at BP, with mean pile height significantly greater in intermediate and deep water compared to shallow (Figure 3C). Across treatments pile heights at all depths reflected differences in water motion between sites (greater intensity at SA as determined by the relative proportion of mobile substrata at each site [Figure S1]): mean heights of all piles in shallow, intermediate and deep depths at BP were 94.1%, 135.9%, and 32.4% taller than those at SA, respectively. Across all depths mean heights of rubble-alone and sponge-rubble piles at BP were also taller than at SA by 21.1% and 49.5%, respectively.

**Figure 4:**
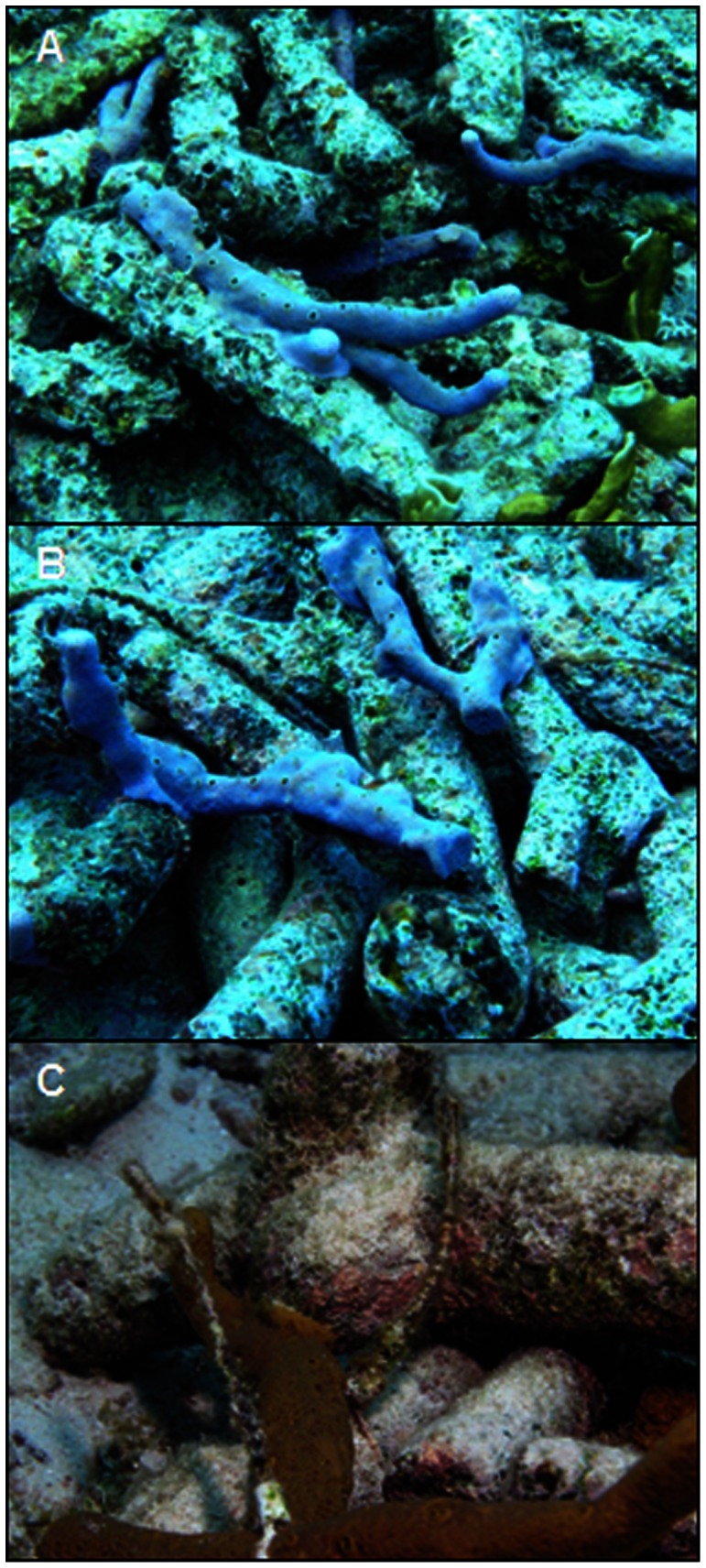
Sponge fragments stabilizing rubble in piles three months post deployment at Sea Aquarium. Sponge fragments inserted into piles grew and adhered to adjacent pieces of rubble in less than three months. A and B. *Aplysina cauliformis* stabilizing sections of rubble piles. C. Coral rubble stabilization by *Aplysina* sp.

Within three months of deployment (October, 2007), sponge fragments inserted into rubble piles at SA had grown in contact with and attached to adjacent pieces of coral rubble (Figure 4). After twelve months, 13 (61.9%) piles of rubble with sponges at SA and 17 (85%) at BP had been temporarily stabilized by the growth and attachment of sponge fragments. Piles of rubble alone were also temporarily stabilized during this period, but by turf algae, which accounted for 4 piles (19%) at SA and 11 piles (55%) at BP. Over four years, 11 piles of rubble alone (52.4%) at SA and 16 piles (80%) at BP were stable in at least one survey due to recruitment and growth of turf algae, macroalgae (e.g., Halimeda), cryptic sponges, and Palythoa. Over this same period, 20 (95.2%) piles of sponge seeded rubble at SA and 19 (95%) at BP were stable in at least one, but often in successive surveys.

**Figure 5:**
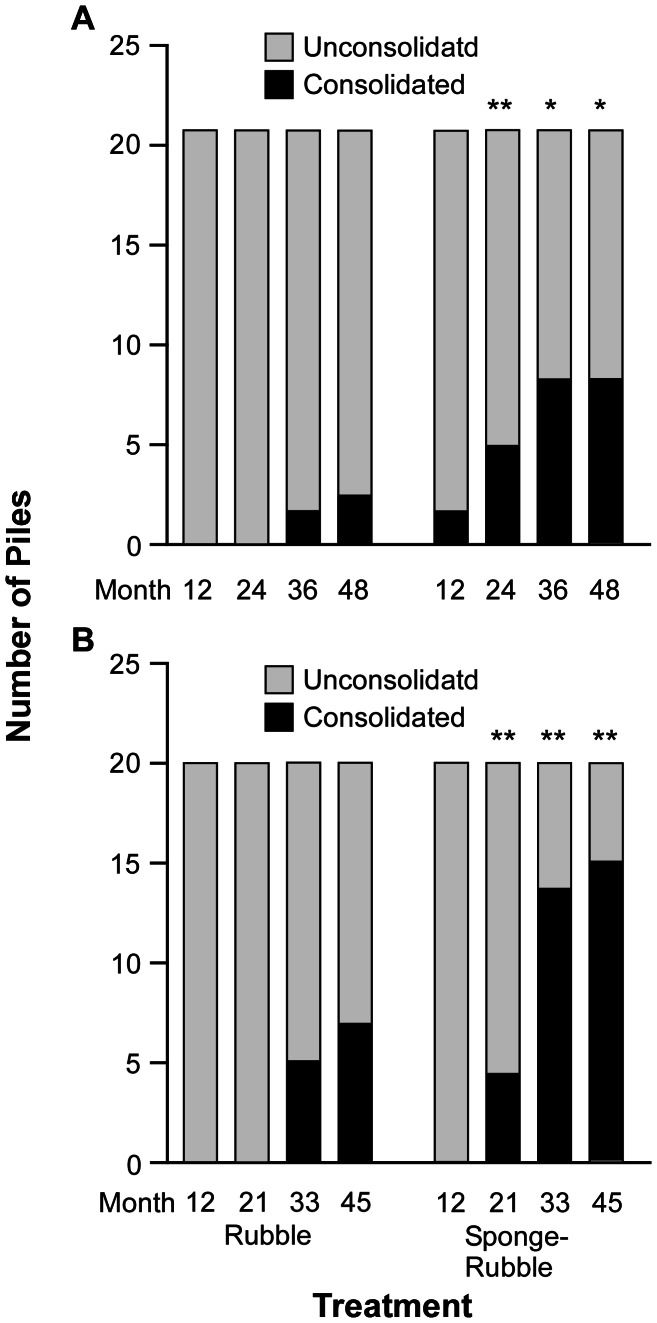
Consolidation of rubble piles with and without sponges by carbonate secreting organisms. A. Sea Aquarium. B. Barracuda point. Number of unconsolidated piles is represented by light grey bars and consolidated piles by black bars. Asterisks above bars indicate significant differences (* *p*<0.05, ** p<0.01) in the proportion of consolidated vs. unconsolidated piles between treatments within the same site, at the same time period, by the G-test of independence. Statistical results from each comparison are provided in Table S3.

Proportions of consolidated vs. unconsolidated rubble piles differed significantly between treatments at both sites over time (Figure 5). By month 24 at SA and 21 at BP, a greater proportion of piles of sponge seeded rubble were consolidated compared to rubble alone (G-tests, df = 1, G = 7.35, P = 0.007, and df = 1, G = 5.99, P = 0.014 for SA and BP, respectively); the same was true for each successive survey (Figure 5, Table S3). In all cases where piles of rubble alone were consolidated, each had been temporarily stabilized during the previous survey by turf algae.

Temporary rubble stabilization did not always lead to consolidation, and replicate piles in both treatments at both sites were lost over time (Table 3). Complete disappearance differed between treatments, and within treatments between sites (Table 3). Rubble pile losses for both treatments were greatest at SA, with 66.5% of all piles lost over the study period compared to 12.5% at BP. Overall, a greater percentage of rubble piles with sponges were present at each site during all surveys (Table 3).

**Table 3:** Percent of coral rubble piles remaining over time per treatment by site.

		Year				
		0	1	2	3	4
Study Site	Treatment	(N)	(%)	(%)	(%)	(%)
Sea Aquarium	Rubble alone	21	85.7	66.7	42.9	23.8
	Sponge-rubble	21	100	85.7	57.1	42.8
Barracuda Point	Rubble alone	20	95	95	85	85
	Sponge-rubble	20	100	100	100	90

Initial number of replicate piles (Year 0) is also given.

Years 1, 2, 3, and 4 correspond to surveys at months 12, 24, 36, and 48 at Sea Aquarium and to surveys at months 12, 21, 33, and 45 at Barracuda Point.

Sponge species did not survive equally well in rubble piles (Table 2). A pattern of reduced relative survival of *Aplysina* sp. (total loss of sponges from their respective piles) was noted after 48 months at SA and 45 months at BP (Table 2). *Aplysina cauliformis* fragments had the highest survival rates.

### Coral Recruitment to Rubble Piles with and without Sponges and Concrete Bound Structures

**Figure 6:**
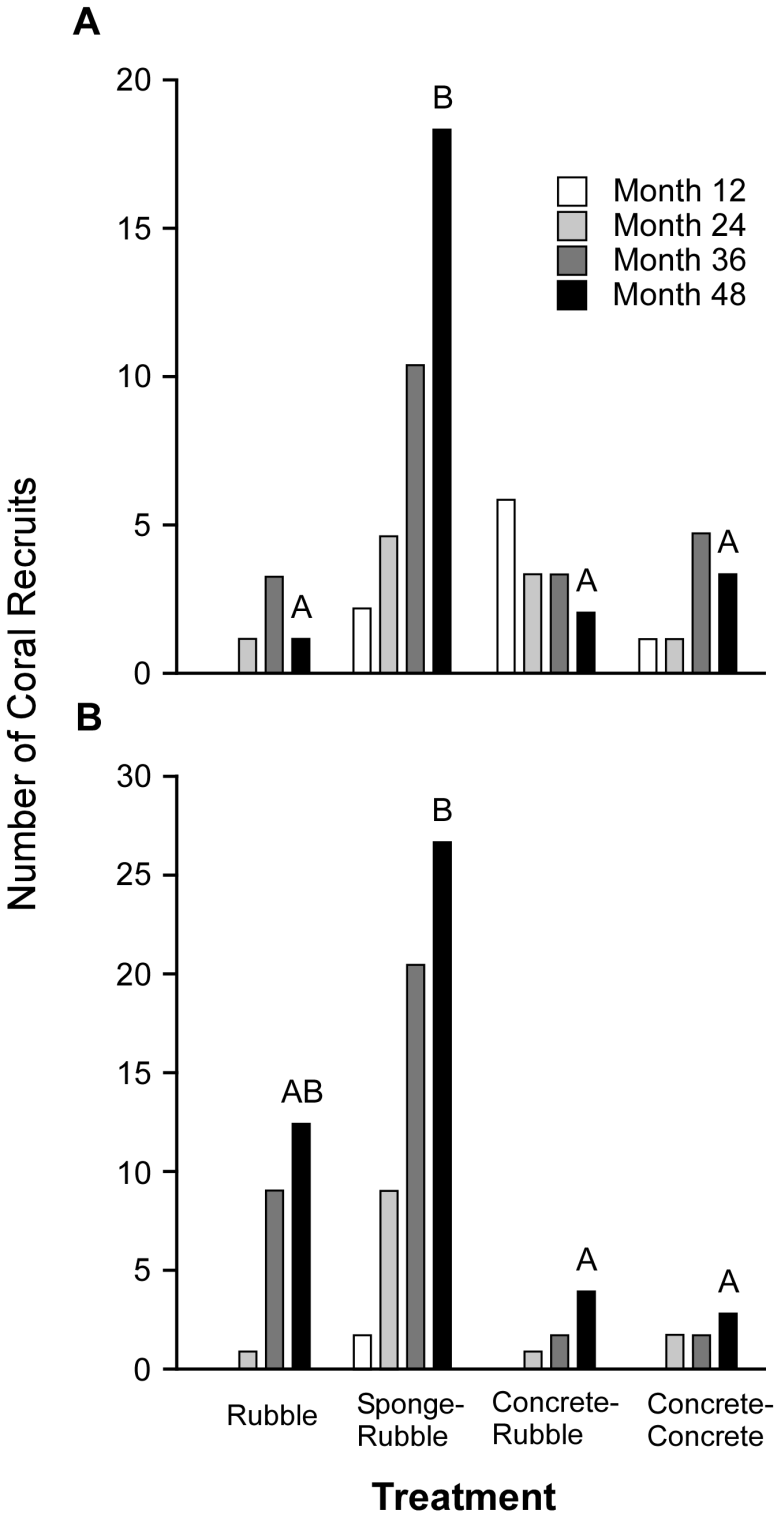
Number of coral recruits at each site, during each survey, by treatment. A. Sea Aquarium. B. Barracuda point. For Sea Aquarium (A): open, light gray, dark gray and black bars indicate the number of coral recruits at months 12, 24, 36, and 48, respectively. For Barracuda Point (B): open, light gray, dark gray and black bars indicate the number of coral recruits at months 12, 21, 33, and 45, respectively. Letters above black bars (months 48 and 45 for Sea Aquarium and Barracuda Point, respectively), indicate significant differences (P<0.05) among treatments within each site in the number of coral recruits based on pairwise comparison with Bonferroni correction. Within each site, bars that share the same letter are not significantly different (P>0.05) from one another.

Recruitment at SA was initially (month 12) greatest to concrete bound coral rubble; however, its replicates lost recruits and gained fewer new recruits in each successive survey, while sponge-rubble continued to accumulate recruits (Figure 6A). By month 24, and in each successive survey, sponge-rubble had greater numbers of recruits than all other treatments. When compared 48 months after deployment, the number of coral recruits at SA differed significantly among treatments (GLM, F _3, 46_ = 10.59, P<0.0001), and a significantly greater number of corals had recruited to sponge-rubble than to all other treatments (Figure 6A). Analyzed over time (repeated measures), treatment itself was not found to significantly influence recruitment (LME, *F*_3, 77_ = 1.55, *P* = 0.208).

Recruitment to all treatments at BP increased through time, and at each survey sponge-rubble had the greatest number of coral recruits (Figure 6B). Number of recruits at BP differed significantly among treatments (GLM, F _3, 67_ = 9.8, P<0.0001) when compared 45 months post deployment. Sponge-rubble had a significantly greater number of recruits than either concrete-rubble or concrete-concrete treatments; however, recruitment did not differ significantly between sponge-rubble and rubble-alone at this site (Figure 6B). Repeated measures analysis revealed that treatment significantly influenced the number of coral recruits at BP over time (LME, F _3, 75_ = 7.77, P = 0.0001), and that a significantly (P<0.05) greater number of corals recruited to sponge-rubble at BP than to all other treatments.

A total of six species of coral were found at the two sites roughly 4 years post deployment (Table 4). *Agaricia agaricites* dominated the number of recruits at both sites, accounting for 95.5% and 71.7% of all recruits at SA and BP, respectively. A distant second, *Porites porites* accounted for 19.5% of recruits at BP, but was not found at SA at month 48. Richness was greatest at BP, with six species represented compared to only two (*A. agaricites* and *Madracis mirabilis*) at SA. At both sites, richness was greatest on sponge-seeded rubble piles (2 of 2 species at SA and 5 of 6 species at BP) (Table 4). Sponge-rubble at BP was the only treatment to which framework building corals recruited, with one recruit each of *Colpophyllia natans* and *Siderastrea siderea* (Table 4).

**Table 4:** Number and identity of coral recruits per treatment 48 and 45 months post deployment at Sea Aquarium and Barracuda Point, respectively.

	Sea Aquarium				Barracuda Point			
Taxa	R	SR	CR	CC	R	SR	CR	CC
Agariciidae								
*Agaricia agaricites*	1	15	2	3	9	18	4	2
Astrocoeniidae								
*Madracis mirabilis*	–	1	–	–	–	1	–	–
Faviidae							–	–
*Favia fragum*	–	–	–	–	1	–	–	–
*Colpophyllia natans*	–	–	–	–	–	1	–	–
Poritidae	–	–	–	–			–	
*Porites porites*	–	–	–	–	3	5	–	1
Siderastreidae	–	–	–	–			–	
*Siderastrea siderea*	–	–	–	–	–	1	–	–

Coral family and species names are given. For treatment: R = Rubble alone, SR = Sponge-rubble; CR = Concrete-rubble; CC = Concrete-concrete “rubble”.

### Sponge Fragment Attachment Rates

Fragments from all three sponge species attached quickly to their individual pieces of coral rubble, many within two days (54%, 21%, and 50% of *A. cauliformis*, *Aplysin*a sp., and *N. erecta* fragments, respectively) and all within four days (Table 5). Number of days taken to attach varied little between fragments, and did not differ significantly among sponge species (ANOVA, F _2, 41_ = 0.1, P = 0.9).

**Table 5:** Number of days taken by sponge fragments to attach to coral rubble.

	Fragments	Volume	Mean	Minimum	Maximum
Sponge species	(N)	(cm^3^)	(days)	(days)	(days)
*Aplysina cauliformis*	11	6.3 (1.81)	2.8 (0.98)	2	4
*Aplysina* sp.	19	10.0 (3.36)	2.9 (0.57)	2	4
*Niphates erecta*	12	18.0 (7.45)	2.8 (0.87)	2	4

Notes: and volume of sponge fragments are given. de (+/−SD)are means (+/− SD). Number and volume of sponge fragments are given. Values for sponge fragment volume and mean attachment rate are means (+/− SD).

### Growth and Tissue Replacement Rates of Donor Sponges

A total of 36, 47, and 45 individuals of *A. cauliformis*, *Aplysina* sp., and *N. erecta*, respectively, were relocated in July, 2008. As predicted, many individuals had lost tissue over the 12 month period, such that mean APC for both *Aplysina* sp. and *N. erecta* was negative (Figure S2). Of the *A. cauliformis*, *Aplysina* sp., and *N. erecta* individuals relocated, 61.1%, 38.3%, and 28.8%, respectively, increased in size between June, 2007 and 2008 (Table 6). Annual percent change for sponges that increased in size differed significantly among species (ANOVA, F _2, 50_ = 5.59, P = 0.006), and *A. cauliformis* increased in size to a significantly greater degree than either *Aplysina* sp. or *N. erecta* (Table 6, Figure S2).

**Table 6:** Annual percent change in volume of sponge individuals that increased in size.

	Individuals	Initial Size	Final Size	APC	Grouping
Sponge Species	(N)	(cm^3^)	(cm^3^)	(%*yr^−1^)	(p<0.05)
*Aplysina cauliformis*	22	92.2 (132.6)	146.3 (182.0)	75.7 (62.5)	A
*Aplysina* sp.	18	914.2 (1112.3)	1331.6 (1786.9)	35.8 (25.7)	B
*Niphates erecta*	13	184.1 (189.8)	218.8 (189.3)	31.2 (25.9)	B

Values for initial and final size as well as APC are means (+/− SD). Significance grouping of species is based on pairwise comparison of mean APC with Bonferroni correction. See Figure S2 for size change of all sponges relocated after 12 months.

**Figure 7:**
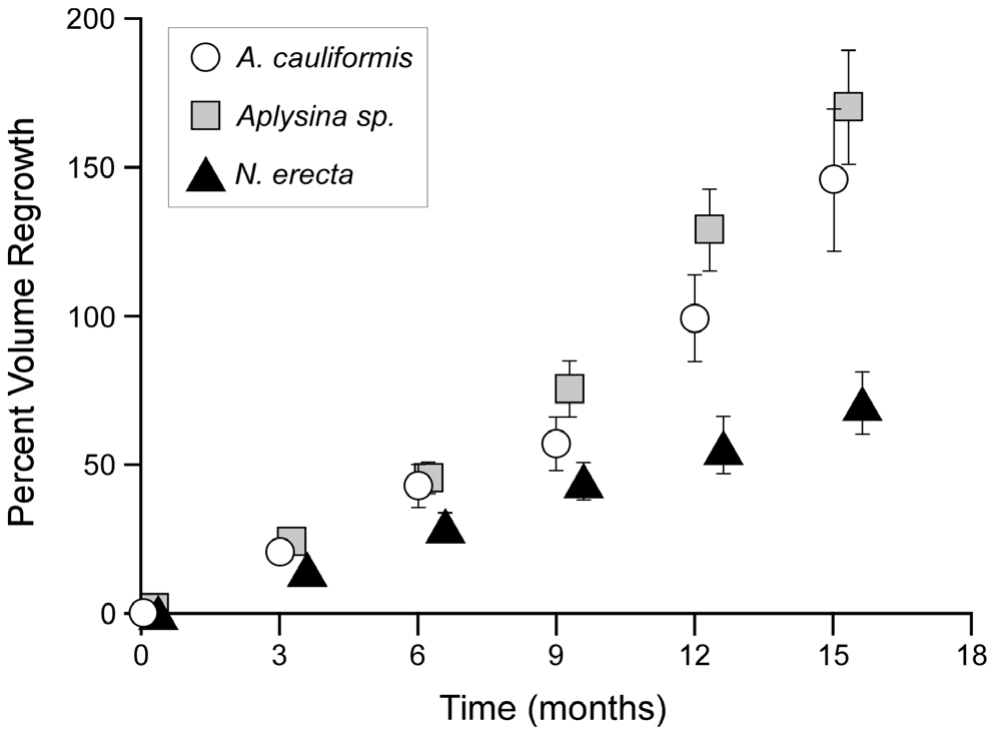
Cumulative replacement of excised tissue (PVR) by sponges. A. Cumulative mean percent of tissue excised that was replaced at each 3 month period, in terms of volume, by all sponges surviving for 15 months: *Aplysina cauliformis (N = 29)*, *Aplysina* sp. (N = 34), *Niphates erecta (N = 39)*. Standard error bars are shown. *Aplysina cauliformis* and *Aplysina* sp. replaced tissue significantly (P<0.05) more rapidly than *N. erecta* (determined by pairwise comparison of means with Bonferroni correction) (See Table S4).

Each species replaced tissue severed from branch tips, though the congeners *A. cauliformis* and *Aplysina* sp. were most adept. Twelve months after excision, *A. cauliformis* and *Aplysina* sp. had, on average, replaced the entire volume of tissue excised (Figure 7). Fifteen months post excision, *N. erecta* individuals had, on average, managed to replace only 71%, and lagged well behind *A. cauliformis* and *Aplysina* sp. (Figure 7). Percent volume replaced (PVR) over the fifteen month period differed significantly among species (LME, F _2, 158_ = 6.85, P = 0.001), with the congeners *A. cauliformis* and *Aplysina* sp. replacing tissue significantly (P<0.05) more rapidly over time than *N. erecta* (Table S4). The interaction between species and log transformed initial volume as well as the fixed effect of log transformed initial volume failed to survive the model selection process.

## Discussion

Physical disturbance from vessel grounding, anchor dragging, and dynamite fishing can result in injury and mortality of reef organisms, damaged reef framework, and sizeable amounts of unstable debris ([17] and references therein). Beds of loose rubble hinder reef recovery [19], and efforts to restore and rehabilitate damaged reefs have used various methods to reconstruct three-dimensional framework, reattach dislodged organisms, and consolidate loose rubble to foster coral recruitment [11]. However, the ecological performance of artificial binding agents and materials used to produce stable substrata have largely not been rigorously evaluated ([36], but see [29], [60] for recent efforts), nor have alternative, natural restoration approaches that could improve outcomes. The series of experiments reported here indicate that: 1) fragments of erect branching coral reef sponges can be used to generate natural, stable substrata suitable for coral recruitment; 2) using natural substrata and binding agents could increase the number and diversity of corals recruiting to restoration sites; 3) harvesting tissue from sponges to seed rubble piles can be sustainable and the use of fragments is largely non-consumptive; 4) sponge species selection is required given species specific differences in performance; and 5) seeding coral rubble with sponges can be an effective tool for assisting the recovery of damaged coral reefs.

### Stabilization and Consolidation of Natural Substrata by Natural Agents

Sponge fragments inserted into piles grew and adhered to adjacent pieces of rubble, stabilizing sections of piles in less than three months (Figure 4). Temporary stabilization by sponges resulted in piles that retained both height and shape over time, across different intensities of water motion to a significantly greater degree than rubble alone (Figure 3B and 3D). Fewer sponge seeded piles were lost to water motion (Table 3), and significantly greater proportions were consolidated by carbonate secreting organisms than were piles of rubble alone (Figure 5). These results corroborate those of Wulff [20], who demonstrated that sponges assist reef rejuvenation by mediating the consolidation of coral skeletons by carbonate secreting organisms; however, distinctly less CCA recruited to rubble in Curaçao, and the rate of consolidation was considerably slower than that reported from Panama. Sponge fragments were therefore required to stabilize rubble in Curaçao far longer than anticipated, but succeeded in doing so. Declining CCA recruitment in Curaçao over the last three decades [61] may explain the difference in consolidation rates. If so, reduced rates of consolidation may not simply be a local concern, but rather a global issue given that elevated carbon dioxide levels have been found to dramatically reduce CCA recruitment and growth [62]. Preliminary rubble stabilization by sponges may, therefore, be more important now than ever.

Piles of coral rubble alone, 14.2% at SA and 35% at BP, were also consolidated by carbonate secreting organisms over the course of the study (Figure 5). In each instance, consolidated piles had been stabilized the previous census by turf algae, macroalgae, cryptic sponges, and Palythoa. This not only reflects the importance of stabilization to the process of consolidation, but the role numerous organisms play in the reincorporation of rubble [42]. Nevertheless, early and continued preliminary stabilization of rubble by implanted sponge fragments resulted in a significantly greater number of consolidated piles over the same period (42.9% and 75% at SA and BP, respectively) (Figure 5).

### Coral Recruitment

Comparison among treatments varying in substratum and binding agent suggest that Caribbean coral larvae preferentially recruit to natural substrata stabilized (e.g., sponges) or consolidated by natural agents (e.g., CCA, bryozoans). Recruitment, in terms of both number and diversity of corals, was greatest to sponge seeded rubble piles (Figure 6, Table 4): 72.7% and 56.5% of all recruits and 5 of 6 species present at months 48 and 45 at SA and BP, respectively, were found on sponge-rubble replicate piles. These results confirm patterns of recruitment from both Atlantic and Pacific reefs. For example, after surveying coral assemblages at two vessel grounding sites (Elpis and Maitland) in the Florida Keys where, three years earlier, different structural restoration plans had been pursued, Miller and Barimo [31] reported that both the abundance and diversity of coral recruits was greater at the Elpis site, where limerock boulders had been deployed instead of concrete structures. In addition, of the corals that recruited to the cast concrete structures at the Maitland site, 60% were found on lime rock chunks embedded in the concrete, which accounted for only 25% of total surface area. Continued investigation of recruitment at these restoration sites again revealed preferential settlement on natural substrata by scleractinians [34]. Reyes and Yap [32] reported that Indo-Pacific corals recruited in greater density to settlement plates made of dead coral vs. concrete or rubber, though differences were not statistically significant. In Dubai, Burt et al. [29] found significantly greater densities of corals recruited to tiles formed of basalt-like gabbro than to concrete, though concrete gained more recruits than terra cotta. However, Gleason and Sutton [60] documented greater recruitment of *Oculina arbuscula* to concrete paving tiles vs. natural surfaces (cleared and uncleared) in the temperate Atlantic, indicating that while preference of larvae for natural substrata appears to hold for many species, it may not extend to all corals in every region.

Similar to restoration sites in the Florida Keys ([24], and see [18]), the brooding coral *A. agaricites* accounted for the majority of recruits (79.4%) at both sites roughly four years post deployment (Table 4). Another brooder, *P. porites,* accounted for 13.2% of total recruits. This pattern is both in line with and provides additional evidence for the general trend of recruitment failure among broadcast spawning, framework building corals in the Caribbean [24], [63], [64]. These functionally important species facilitate reef community development through habitat formation [65]. It is therefore meaningful that, of the four treatments at both study sites, framework building corals only recruited to sponge seeded coral rubble (Table 4). Interestingly, Vermeij et al. [33] documented a preference, under normal environmental conditions, for limestone and crustose coralline algae by the larvae of the Caribbean framework builder *Montastraea faveolata*. In opposition to findings reported here, Schittone [34] documented a significant trend for the larvae of *S. siderea* to settle on concrete at a restoration site in the Florida Keys; however, its congener, *S. radians*, notably settled solely on natural substrata. Further investigation of preference and survival of framework building coral larvae in relation to natural substrata and binding agents is clearly needed, and would only improve our ability to aid reef recovery. Undoubtedly concrete has its uses, but patterns of recruitment reported here and in the literature suggest that alternative methods of establishing consolidated, natural substrata could yield greater numbers and diversity of coral recruits at restoration sites.

### Harvesting Sponge Fragments Sustainably and Growing Tissue in Nurseries

On average, *A. cauliformis* and *Aplysina* sp. replaced 100% of the volume of tissue excised to seed rubble piles within 12 months, while *N. erecta* replaced 71% of the volume of tissue removed in 15 months (Figure 7). This suggests single 10 cm long fragments could be sustainably harvested from individuals at roughly annual intervals, depending on the species. Individuals of *A. cauliformis, Aplysina* sp. and *N. erecta* increased in size over 12 months by 75.7%, 35.8%, and 31.2%, respectively (Table 6). These rates are in line with those of uncaged *A. cauliformis* and *N. erecta* fragments in the Florida Keys (e.g., 74% and 33%, respectively) [66], but are rapid compared to the roughly 12% per year increase reported for massive and globular shaped tropical reef sponges ([67] and references therein). Conservative estimates suggest that if 50% of the total volume of an individual were harvested to seed rubble piles, it would take roughly 1.5 to 3.5 years to replace, depending on the species. Furthermore, given that erect branching sponges fragment naturally (Figure S2A) [46], [47], using fragments to seed rubble simply approximates dispersal, and likely increases sponge biomass on the reef overall. Depending on the number and size of erect branching sponges at damaged sites, tips could be harvested from individuals and grown in sponge “nurseries” to increase the amount of biomass available for restoration [68]; as has been done for coral species (e.g., [69], [70]).

### Sponge Species Selection for Restoration

Selection of appropriate species to use in restoration has been discussed widely in the literature (e.g., [71]–[73]), and specific traits have been identified that can aid discrimination between alternatives. For example, life history traits have been successfully used to improve restoration of seagrass meadows [74], and asexual reproduction has been linked to success, particularly in early stages of restoration [73]. Asexual reproduction dominates the life histories of erect branching sponges, and fragments of all three species attached rapidly to pieces of coral rubble, often in as little as two days (Table 5); rates in line with other erect branching Caribbean species ([20], [46], B.C. Biggs unpublished data). However, comparison among species in: 1) rates of replacement of excised tissue (Figure 7); 2) annual percent change in volume (Table 6); and 3) patterns of survival in rubble piles (Table 2) as well as propensity to stabilize rubble revealed noted differences in performance. Overall, *A. cauliformis* performed best, with high survival in piles and rapid growth and tissue replacement. Its congener, *Aplysina* sp., replaced greater than 100% of its excised tissue in 12 months, but grew relatively slowly and survived poorly in rubble piles. *Niphates erecta* grew and replaced tissue most slowly, and fragments survived poorly on the outsides of rubble piles, but well on the insides. Clearly, species selection is worth considering when using this technique.

Our ability to predict which species might most successfully be used in restoration is hampered by our limited knowledge of many basic aspects of sponge biology. Given the impracticality of testing all species, efforts are underway to identify key traits that would allow generation of a list of species that could be reliably used. For the time being, successful outcomes might most dependably be attained by using multi-species assemblages of erect branching reef sponges. Mutualism appears to be the rule among these sponges ([75], [76], but see [77] for exploitation of mutualism), and species with different skeletal characteristics [48], [78], [79] and degrees of susceptibility to consumption by predators [80], [81] could be combined to increase survival and growth of fragments in rubble piles.

Care should still be exercised when mixing sponges, as not all species will be advantageous. For instance, in shallow reef waters the bioeroding sponge *Cliona (Anthosigmella) varians*, which typically exhibits an encrusting morphology (forma *incrustans*), occasionally takes a branching form (forma *rigida*) similar to that of forma *varians* in bays and lagoons [82], [83]. The often erect appearing *Desmapsamma anchorata* would also not be a suitable choice. Upon closer examination, the sponge can be found to be encrusting and thereby smothering its hosts (e.g., erect branching sponges and gorgonians) [77]. Its flimsy skeleton is bolstered by the organisms it encrusts, allowing it to invest heavily in tissue production; however, its rapid growth is offset by its rate of mortality, especially on rigid substrata [77]. Thus, while *D. anchorata* has been observed overgrowing corals, its existence at any one site is usually ephemeral. In general, such overgrowths are far less common than are standoffs among sponge-coral interactions [84], especially when observed repeatedly over time [85]. Although reports of negative interactions have generated concern, in actuality only a handful of sponge species have been observed to overgrow and kill corals (see [86] for a review); fewer still have been demonstrated to do so by chemical means (e.g., *Plakortis halichondrioides* [87] and *Siphonodictyon coralliphagum* [88] in the Caribbean). Most confirmed cases of sponges overwhelming corals on reefs have involved encrusting or bioeroding species, particularly those with photosynthetic endosymbionts, and often in situations in which corals have been stressed (reviewed by [86]). Nonetheless, when such circumstances arise, the effects can be significant. Counterintuitively, sponge attachment can often benefit corals; for example, increasing their survival by an order of magnitude [89]. Thus, while attachment by sponges can result in small amounts of tissue loss, the net benefits accrued by corals would appear in most circumstances to far outweigh the costs.

### Management Implications

Sponges are important members of coral reef communities, performing functions critical to both reef maintenance and resilience. Often surpassing Caribbean corals in numbers of species, individuals, and volume of living tissue, sponges account for a considerable share of reef biodiversity and biomass [41], [90]. Their large sizes and elaborate morphologies contribute to habitat complexity, and many organisms take shelter in or around these filter feeders [90], [91]. Sponge attachment lessens the chance of coral mortality via toppling [89], and encrustation of exposed carbonate bases protects corals from bioeroding organisms [92]. Efficient removal by sponges of bacteria and particulate organic matter from the water column [93], [94] helps maintain reef water clarity, and provides a critical coupling between primary production in the water column and the benthic community [95]. Additionally, prevention of disease immediately following physical disturbances (e.g., hurricanes, boat groundings) may depend on the removal of potentially harmful bacteria by sponges [41]. Using sponge fragments to seed piles of rubble returns these functional roles to damaged reefs while also increasing biodiversity and biomass, all desirable elements in habitat restoration [11], [16], [72], [96], [97].

Compared to using concrete, rubble stabilization by sponges and consolidation by carbonate secreting organisms are dynamic processes, the autocatalytic natures of which mean that rubble piles can naturally increase in size over time. For example, many shallow water corals at SA were damaged following tropical storm Omar in 2008 (personal observation), and newly generated pieces of rubble were found incorporated (by sponge attachment) into experimentally generated piles in July, 2009. Omar also tore apart some of the shallowest sponge-rubble piles at SA, but sections of these piles were found attached to the benthos less than two meters from their initial positions in July, 2009. These sections had been held together by their sponges, and rendered motionless by sponge attachment to the benthos; rubble piles without sponges simply disappeared.

Site and injury specific factors will likely determine the success of using sponges to facilitate rubble consolidation (e.g., water depth and water motion; degree, extent and type of reef damage; availability of sponges). Loss of some sponge seeded rubble piles is to be expected, especially in shallow, heavily wave swept areas. However, many piles in relatively shallow water survived tropical storms Felix in 2007 and Omar in 2008 (Table 3), suggesting even a high degree of episodic water motion may be tolerable prior to consolidation. Furthermore, despite losses of piles, recruitment to sponge-rubble far outweighed that to all other treatments (Figure 6, Table 4), suggesting time and effort are well spent seeding rubble with sponge fragments. Successful use may also depend on the size that rubble piles are made. Given that erect branching sponges generally reside on exposed reef surfaces, it seems they would perform best near pile surfaces; thus, constructing piles larger than a meter squared would not be advised.

Many techniques used to create stable substrata and topographical complexity are labor intensive and require considerable sums of money for materials and equipment; factors that hinder their use in developing countries [17]. Low-cost, low-tech methods applicable to the developing world have generally received little attention (but see [98], [99]), though they are cited as important research goals in reef restoration [17], [36]. Seeding rubble with sponge fragments offers such an alternative, and can be used to restore small scale reef damage at local levels: a few hours spent snorkeling or diving with a razor blade can yield numerous sponge fragments ready for use in seeding small piles of rubble formed on nearby reefs, and nylon zip ties can be replaced with cotton string to secure sponge fragments to pieces of rubble. Given the amount of damage associated with even small vessel groundings [51], and their widespread occurrence [11, 50, and references therein], using this simple and inexpensive approach could result in a great many more sites being restored in ecologically successful ways (i.e., increased coral recruitment, biodiversity and functional roles).

Widespread habitat degradation coupled with our dependence on ecosystem derived goods and services [100]–[102] have driven interest in conservation and management. Compensatory approaches are increasingly being relied upon to rehabilitate and restore degraded habitat; however, success in restoring structure and function are far less common than failure [11], [28]. Progress will best be made through experimentation and rigorous evaluation of alternative approaches, and shifting the balance may require incorporating natural processes that facilitate recovery into our restoration toolkit. Methods will necessarily need to be tailored specifically to the biology of the system under investigation [74]; however, as demonstrated here for coral reefs as well as in other systems, harnessing organisms that jump start successional pathways and facilitate recovery can significantly improve restoration outcomes [28], [37]–[40].

## Supporting Information

Figure S1 **Proportional representation of substratum type by reef study site.** Bar plots show percent of total substratum represented by each substratum type (pavement, live coral, dead coral, coral rubble, and sand) at each site. Data are from AGRRA surveys and represent combined information from 4 separate transects (10 m long each) surveyed between the depths of 4.5 and 13.7 m at each site (N = 8 total transects, 4 transects per site). Substratum type was recorded for.25 m^2^ quadrats placed at 2 m intervals (starting at meter 1) along each transect surveyed (N = 40 quadrats in total, 20 quadrats per site, 5 quadrats per transect). Substratum type may be divided into mobile (e.g., coral rubble and sand) and immobile (pavement, live coral and dead coral) substrata. AGRRA surveys suggest difference in substratum composition between sites: mobile substrata (e.g., coral rubble and sand) accounted for 35% of substrata at BP but only 15% at SA. Reduced accumulation of mobile substrata at SA suggest greater intensity of water motion as compared to BP.
(PDF)

Figure S2 **Mean annual percent change in sponge size.** A. Mean annual percent change in volume for all surviving sponges (N = 36, 47, and 45 for *A. cauliformis*, *Aplysina* sp., and *N. erecta*, respectively). B. Mean annual percent change in volume for those sponges that increased in size over the 12 month period (N = 22, 18, and 13 for *A. cauliformis*, *Aplysina* sp., and *N. erecta*, respectively). Bars represent SE. Light gray diamonds indicate *Aplysina cauliformis*, dark gray squares indicate *Aplysina* sp. and black triangles indicate *Niphates erecta*. Many individuals had lost tissue over the 12 month period, such that mean percent size change for all surviving *Aplysina* sp. and *N. erecta* individuals was negative (A). For those sponges that increased in size between June, 2007 and 2008, *Aplysina cauliformis* grew significantly more (P<0.05) than either *Aplysina* sp. or *N. erecta* (B).
(PDF)

Table S1 **Proportional representation of coral species by site.** Coral family and species names, coral morphology, number of individuals per site, percent of total number of individuals per site as well as totals for each of the previous categories are given for Sea Aquarium and Barracuda Point reef sites, respectively. Numbers of individuals at each census site represent combined information from 4 separate transects (10 m long each) surveyed between the depths of 4.5 and 13.7 m (N = 8 total transects, 4 transects per site). A total of 17 species of coral were encountered at the two sites, 15 at Sea Aquarium and 13 at Barracuda Point; 12 coral species were shared between the two sites. With few exceptions, numbers of individuals per species and percent of total individuals per species were similar among those species encountered at both census sites. Additionally, numbers of individuals per morphological group and percent of total individuals per morphological group are highly similar at both sites (see Table S2).
(PDF)

Table S2 **Proportional representation of coral morphological groups by site.** Coral morphology, number of individuals per morphological group, percent of total number of individuals per morphological group as well as totals for each of the previous categories are given for Sea Aquarium and Barracuda Point reef sites, respectively. Numbers of individuals represent combined information from 4 separate transects (10 m long each) surveyed at each census site between the depths of 4.5 and 13.7 m. See Table S1 for species in each morphological group. Of the four morphological groups, only branching corals (e.g., *Acropora palmata*) were not encountered in the surveys at both sites; however, both *A. palmata* and its congener *A. cervicornis* are present at each site (personal observation). Numbers of individuals per morphological group and percent of total individuals per group are similar between sites. Massive corals, which include many framework building species, are not only similar with respect to their proportional representation at each site, but are also the dominant morphological group at both sites, in terms of both numbers of individuals and percent of total number of individuals.
(PDF)

Table S3 **Rubble pile consolidation over time by treatment and site.** Differences in the proportion of consolidated vs. unconsolidated piles between treatments (rubble alone vs. sponge-rubble) within the same site, at the same time period were investigated using the G-test of independence. Site, monitoring period, and results of G-tests are given for each comparison. P-values in bold represent significant differences in the proportion of consolidated and unconsolidated piles between treatments.
(PDF)

Table S4 **Sponge tissue replacement (PVR) over 15 months.** Number of individuals and percent of total individuals (initial number) remaining after 15 months are given. Values for volume of tissue excised, volume of tissue replaced after 15 months, and percent volume of tissue replaced (PVR) after 15 months are means (+/− SD). Percent volume replaced (PVR) over time (repeated measures) was analyzed using LME; sponge species (fixed effect) was significant. Significance grouping of species based on pairwise comparison of mean PVR over time with Bonferroni correction is given. The congeners *A. cauliformis* and *Aplysina* sp. replaced the volume of tissue excised significantly more rapidly than *N. erecta*.
(PDF)

Text S1 **Supporting information for Linear Mixed-Effects Models.**
(PDF)
