# Supplementary figures and images for: Harnessing Natural Recovery Processes to Improve Restoration Outcomes: An Experimental Assessment of Sponge-Mediated Coral Reef Restoration
Source: PLoS One. 2013 Jun 4;8(6):e64945. doi: 10.1371/journal.pone.0064945 (PMC3672152; doi:10.1371/journal.pone.0064945)

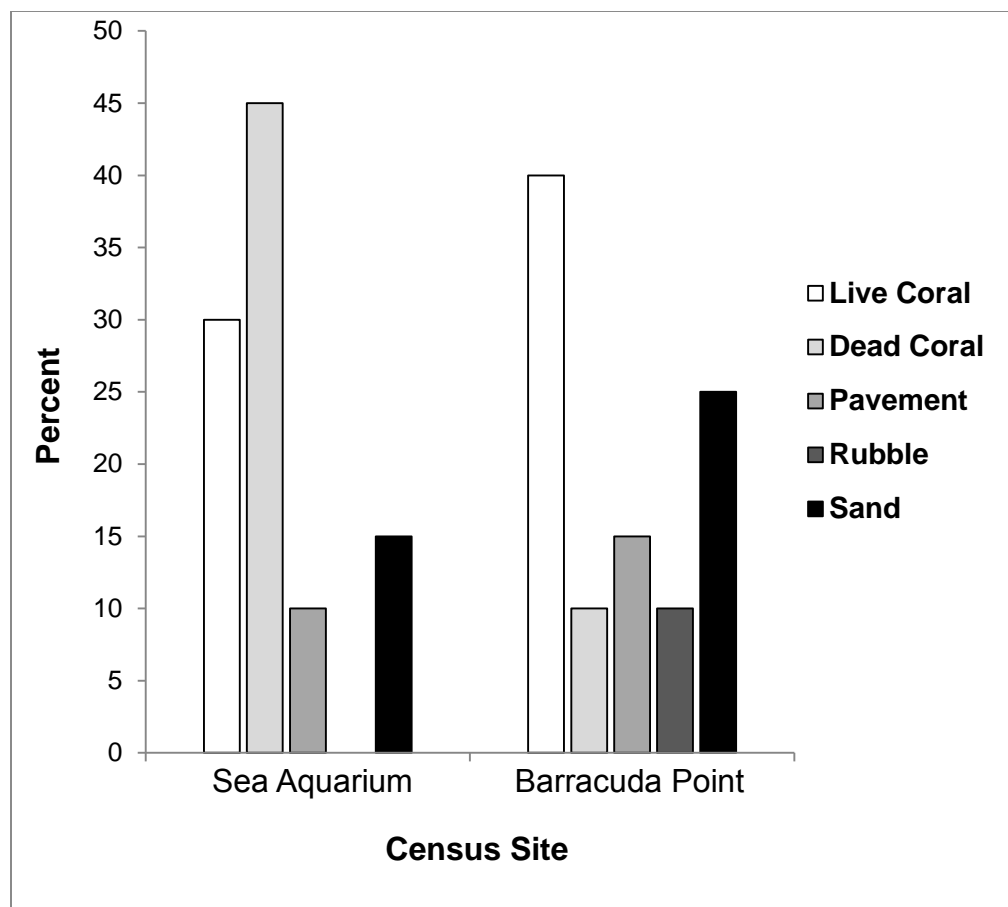

**Figure S1: Proportional representation of substratum type by reef study site.**

Supplement: Figure S1 — Proportional representation of substratum type by reef study site. Bar plots show percent of total substratum represented by each substratum type (pavement, live coral, dead coral, coral rubble, and sand) at each site. Data are from AGRRA surveys and represent combined information from 4 separate transects (10 m long each) surveyed between the depths of 4.5 and 13.7 m at each site (N = 8 total transects, 4 transects per site). Substratum type was recorded for.25 m2 quadrats placed at 2 m intervals (starting at meter 1) along each transect surveyed (N = 40 quadrats in total, 20 quadrats per site, 5 quadrats per transect). Substratum type may be divided into mobile (e.g., coral rubble and sand) and immobile (pavement, live coral and dead coral) substrata. AGRRA surveys suggest difference in substratum composition between sites: mobile substrata (e.g., coral rubble and sand) accounted for 35% of substrata at BP but only 15% at SA. Reduced accumulation of mobile substrata at SA suggest greater intensity of water motion as compared to BP. (PDF) [file pone.0064945.s001.pdf]

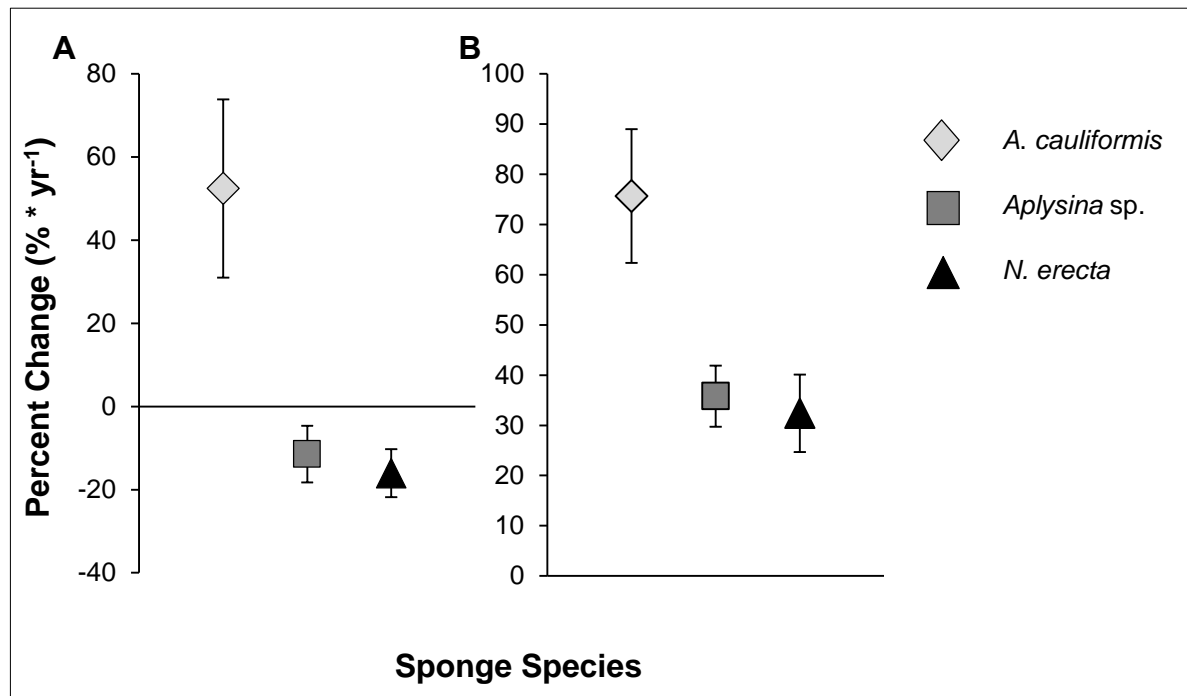

Figure S2: Mean annual percent change in sponge size.

Supplement: Figure S2 — Mean annual percent change in sponge size. A. Mean annual percent change in volume for all surviving sponges (N = 36, 47, and 45 for A. cauliformis, Aplysina sp., and N. erecta, respectively). B. Mean annual percent change in volume for those sponges that increased in size over the 12 month period (N = 22, 18, and 13 for A. cauliformis, Aplysina sp., and N. erecta, respectively). Bars represent SE. Light gray diamonds indicate Aplysina cauliformis, dark gray squares indicate Aplysina sp. and black triangles indicate Niphates erecta. Many individuals had lost tissue over the 12 month period, such that mean percent size change for all surviving Aplysina sp. and N. erecta individuals was negative (A). For those sponges that increased in size between June, 2007 and 2008, Aplysina cauliformis grew significantly more (P<0.05) than either Aplysina sp. or N. erecta (B). (PDF) [file pone.0064945.s002.pdf]
