# Supplementary material for: Harnessing Natural Recovery Processes to Improve Restoration Outcomes: An Experimental Assessment of Sponge-Mediated Coral Reef Restoration
Source: PLoS One. 2013 Jun 4;8(6):e64945. doi: 10.1371/journal.pone.0064945 (PMC3672152; doi:10.1371/journal.pone.0064945)
Supplement: Table S1 — Proportional representation of coral species by site. Coral family and species names, coral morphology, number of individuals per site, percent of total number of individuals per site as well as totals for each of the previous categories are given for Sea Aquarium and Barracuda Point reef sites, respectively. Numbers of individuals at each census site represent combined information from 4 separate transects (10 m long each) surveyed between the depths of 4.5 and 13.7 m (N = 8 total transects, 4 transects per site). A total of 17 species of coral were encountered at the two sites, 15 at Sea Aquarium and 13 at Barracuda Point; 12 coral species were shared between the two sites. With few exceptions, numbers of individuals per species and percent of total individuals per species were similar among those species encountered at both census sites. Additionally, numbers of individuals per morphological group and percent of total individuals per morphological group are highly similar at both sites (see Table S2). (PDF) [file pone.0064945.s003.pdf]

Table S1. Proportional representation of coral species by site.

			Sea Aquarium		Barracuda Point	
**Family**	**Species**	**Morphology**	**N**	**% Total N**	**N**	**% Total N**
Acroporidae	*Acropora palmata*	Branching	0	0	1	2.1
Agariciidae	*Agaricia agaricites*	Leafy	4	8.7	5	10.4
Astrocoeniidae	*Madracis mirabilis*	Fingers	7	15.2	5	10.4
Faviidae	*Colpophyllia natans*	Massive	4	8.7	3	6.3
	Diploria labyrinthiformis	Massive	1	2.2	1	2.1
	Diploria strigosa	Massive	4	8.7	3	6.3
	Montastraea annularis	Massive	4	8.7	6	12.5
	Montastraea cavernosa	Massive	5	10.9	9	18.8
	Montastraea faveolata	Massive	3	6.5	2	4.2
	Solenastrea bournoni	Massive	1	2.2	0	0
Meandrinidae	*Dendrogyra cylindrus*	Massive	2	4.3	0	0
	Eusmilia fastigiata	Fingers	3	6.5	2	4.2
	Meandrina meandrites	Massive	1	2.2	1	2.1
Poritidae	*Porites astreoides*	Massive	1	2.2	2	4.2
	Porites porites	Fingers	3	6.5	0	0
Siderastreidae	*Siderastrea siderea*	Massive	3	6.5	8	16.7
		**Total**	46	100	48	100
