# Supplementary material for: Harnessing Natural Recovery Processes to Improve Restoration Outcomes: An Experimental Assessment of Sponge-Mediated Coral Reef Restoration
Source: PLoS One. 2013 Jun 4;8(6):e64945. doi: 10.1371/journal.pone.0064945 (PMC3672152; doi:10.1371/journal.pone.0064945)
Supplement: Table S2 — Proportional representation of coral morphological groups by site. Coral morphology, number of individuals per morphological group, percent of total number of individuals per morphological group as well as totals for each of the previous categories are given for Sea Aquarium and Barracuda Point reef sites, respectively. Numbers of individuals represent combined information from 4 separate transects (10 m long each) surveyed at each census site between the depths of 4.5 and 13.7 m. See Table S1 for species in each morphological group. Of the four morphological groups, only branching corals (e.g., Acropora palmata) were not encountered in the surveys at both sites; however, both A. palmata and its congener A. cervicornis are present at each site (personal observation). Numbers of individuals per morphological group and percent of total individuals per group are similar between sites. Massive corals, which include many framework building species, are not only similar with respect to their proportional representation at each site, but are also the dominant morphological group at both sites, in terms of both numbers of individuals and percent of total number of individuals. (PDF) [file pone.0064945.s004.pdf]

Table S2. Proportional representation of coral morphological groups by site.

	Sea Aquarium		Barracuda Point	
**Morphology**	**N**	**% Total N**	**N**	**% Total N**
Branching	0	0	1	2.1
Fingers	13	28.3	7	14.6
Leafy	4	8.7	5	10.4
Massive	29	63	35	72.9
**Total**	**46**	**100**	**48**	**100**
