# Supplementary material for: Harnessing Natural Recovery Processes to Improve Restoration Outcomes: An Experimental Assessment of Sponge-Mediated Coral Reef Restoration
Source: PLoS One. 2013 Jun 4;8(6):e64945. doi: 10.1371/journal.pone.0064945 (PMC3672152; doi:10.1371/journal.pone.0064945)
Supplement: Table S3 — Rubble pile consolidation over time by treatment and site. Differences in the proportion of consolidated vs. unconsolidated piles between treatments (rubble alone vs. sponge-rubble) within the same site, at the same time period were investigated using the G-test of independence. Site, monitoring period, and results of G-tests are given for each comparison. P-values in bold represent significant differences in the proportion of consolidated and unconsolidated piles between treatments. (PDF) [file pone.0064945.s005.pdf]

Table S3: Rubble pile consolidation over time by treatment and site.

	Period	G-test of Independence		
**Site**	**(month)**	***df***	***G***	***p***
Sea Aquarium	12	1	2.873	0.09
	24	1	7.609	**0.006**
	36	1	6.443	**0.011**
	48	1	4.348	**0.037**
Barracuda Point	12	1	0	1
	21	1	7.648	**0.006**
	33	1	8.424	**0.004**
	45	1	6.661	**0.009**
