# Supplementary material for: Harnessing Natural Recovery Processes to Improve Restoration Outcomes: An Experimental Assessment of Sponge-Mediated Coral Reef Restoration
Source: PLoS One. 2013 Jun 4;8(6):e64945. doi: 10.1371/journal.pone.0064945 (PMC3672152; doi:10.1371/journal.pone.0064945)
Supplement: Table S4 — Sponge tissue replacement (PVR) over 15 months. Number of individuals and percent of total individuals (initial number) remaining after 15 months are given. Values for volume of tissue excised, volume of tissue replaced after 15 months, and percent volume of tissue replaced (PVR) after 15 months are means (+/− SD). Percent volume replaced (PVR) over time (repeated measures) was analyzed using LME; sponge species (fixed effect) was significant. Significance grouping of species based on pairwise comparison of mean PVR over time with Bonferroni correction is given. The congeners A. cauliformis and Aplysina sp. replaced the volume of tissue excised significantly more rapidly than N. erecta. (PDF) [file pone.0064945.s006.pdf]

Table S4. Sponge tissue replacement (PVR) over 15 months.

Sponge Species	Individuals (N)	(%)	Volume Excised (cm^3)	Volume Replaced (cm^3)	PVR (%)	Grouping ($p<0.05$)
Aplysina cauliformis	29	48.3	12.7 (5.8)	17.7 (17.4)	150.9 (124.5)	A
Aplysina sp.	34	57.6	38.1 (13.9)	60.8 (41.5)	171.1 (116.9)	A
Niphates erecta	39	63.9	33.1 (27.7)	18.3 (13.4)	71.0 (62.28)	B
