# Supplementary material for: Harnessing Natural Recovery Processes to Improve Restoration Outcomes: An Experimental Assessment of Sponge-Mediated Coral Reef Restoration
Source: PLoS One. 2013 Jun 4;8(6):e64945. doi: 10.1371/journal.pone.0064945 (PMC3672152; doi:10.1371/journal.pone.0064945)
Supplement: Text S1 — Supporting information for Linear Mixed-Effects Models. (PDF) [file pone.0064945.s007.pdf]

Text S1. Supporting information for Linear Mixed-Effects Models.

During Linear mixed-effects model (LME) random components optimization, a correlation structure was included in the model if autocorrelation plots of normalized residuals suggested violation of independence; a variance structure that allowed for different variance per stratum was included if plots of the normalized residuals vs. explanatory variables revealed heterogeneity of variance. In all cases, random components optimization involved a top down model selection process whereby nested models were compared using Akaike information criterion to determine the optimal correlation and variance structure for each minimally adequate model.

With respect to LME models for the heights of rubble piles at each site over time, optimization of the random effects portion of the model required that an auto-regressive moving average (ARMA) correlation structure, with no autoregressive parameters and one moving average parameter be included in the model for SA to remove residual auto correlation. For BP, an ARMA correlation structure with two autoregressive parameters and no moving average parameters as well as a variance structure allowing different variance per depth were both required to obtain the optimal random effects structure.

Regarding the LME models for Differences in recruitment among treatments over time at each site, optimization of the random effects portions of the minimally adequate models of coral recruitment for both SA and BP required that a compound symmetrical correlation structure as well as a variance structure allowing different variance per treatment be included in the models.
